# Online Prediction of Physico-Chemical Quality Attributes of Beef Using Visible—Near-Infrared Spectroscopy and Chemometrics

**DOI:** 10.3390/foods8110525

**Published:** 2019-10-23

**Authors:** Amna Sahar, Paul Allen, Torres Sweeney, Jamie Cafferky, Gerard Downey, Andrew Cromie, Ruth Hamill M.

**Affiliations:** 1 Teagasc Food Research Centre Ashtown, Dublin D15 KN3K, Ireland; Amna.sahar@teagasc.ie (A.S.); paul.allen@teagasc.ie (P.A.); jamie.cafferky@teagasc.ie (J.C.); Gerard.Downey@teagasc.ie (G.D.); 2 UCD School of Veterinary Medicine, University College Dublin, Belfield, Dublin D04 W6F6, Ireland; torres.sweeney@ucd.ie; 3 Irish Cattle Breeders Federation, Highfield House, Shinagh, Bandon, Co. Cork P72 X050, Ireland; acromie@icbf.com

**Keywords:** meat, quality, near-infrared spectroscopy, on-line, monitoring

## Abstract

The potential of visible–near-infrared (Vis–NIR) spectroscopy to predict physico-chemical quality traits in 368 samples of bovine musculus longissimus thoracis et lumborum (LTL) was evaluated. A fibre-optic probe was applied on the exposed surface of the bovine carcass for the collection of spectra, including the neck and rump (1 h and 2 h post-mortem and after quartering, i.e., 24 h and 25 h post-mortem) and the boned-out LTL muscle (48 h and 49 h post-mortem). In parallel, reference analysis for physico-chemical parameters of beef quality including ultimate pH, colour (L, a*, b*), cook loss and drip loss was conducted using standard laboratory methods. Partial least-squares (PLS) regression models were used to correlate the spectral information with reference quality parameters of beef muscle. Different mathematical pre-treatments and their combinations were applied to improve the model accuracy, which was evaluated on the basis of the coefficient of determination of calibration (R^2^C) and cross-validation (R^2^CV) and root-mean-square error of calibration (RMSEC) and cross-validation (RMSECV). Reliable cross-validation models were achieved for ultimate pH (R^2^CV: 0.91 (quartering, 24 h) and R^2^CV: 0.96 (LTL muscle, 48 h)) and drip loss (R^2^CV: 0.82 (quartering, 24 h) and R^2^CV: 0.99 (LTL muscle, 48 h)) with lower RMSECV values. The results show the potential of Vis–NIR spectroscopy for online prediction of certain quality parameters of beef over different time periods.

## 1. Introduction

A wide range of factors interactively affect the quality of meat, including sex, genotype, rearing conditions, feeding practices, transport, slaughtering and post-mortem handling of the carcass. Meat quality is a complex set of parameters including physico-chemical, chemical and sensory quality. Quality parameters like aroma, flavour, mouth-feel and tenderness can be evaluated by sensory analysis. In addition to these traits, other quality attributes such as colour, water-holding capacity, texture and pH can be studied using instrumental techniques. These technological traits are extremely important as they provide data on the development of ultimate meat quality and also convey information on appreciation of the product and its value, providing specific and important evidence on overall meat quality as it varies among individuals of a population [1]. Meat colour is important to the consumer as a key cue in perception at the point of sale and, therefore, has a major bearing on the decision to purchase [2]. Drip loss is exudate lost from meat through cutting, heating and pressing [3], and losses of ~5% are common in beef [2]. Water-holding-capacity traits such as drip loss and cook loss are important, as they represent variability in economic losses for the processor and furthermore, nutritional losses for the consumers [4]. Variation in pH fall and ultimate pH during the conversion of muscle to meat influence water-holding capacity, colour, and, through influencing the ultimate contractile state and proteolytic enzyme activity post-mortem, tenderness.

In order to improve the overall population for meat quality, through incorporation of meat quality into breeding programmes, a means of providing information on quality, routinely and for as many animals as possible, is required. The assessment of technological meat quality traits using conventional approaches is disadvantageous in terms of time consumption, sample destruction, sample preparation, requirement of expert analysts, chemical utilization and lack of on-site facilities in processing plants for detailed quality evaluation, which adds to operational costs. Subsequently, these traditional methods lack the potential to be applied online for the prediction of meat quality attributes in the industry [5]. It is therefore required to introduce some rapid, non-invasive, non-destructive, chemical-free and more reliable approaches for accurate and online determination of meat quality, which enables quality assessment based on a multivariate approach. 

Non-destructive methods include spectroscopic techniques, use of biosensors, electronic noses, ultrasound methods, microscopy, microwave characterization, nuclear magnetic resonance and dielectric methods [6]. Among these approaches, spectroscopic methods have gained significant popularity regarding the prediction of numerous quality attributes of meat in the last decade. In the case of spectroscopy, electromagnetic radiations in the ultraviolet, visible, near-, mid- and far-infrared regions interact with matter, providing fingerprints of the samples under consideration, which can further be processed to extract useful qualitative and quantitative information [7]. Globally, spectroscopic methods have been extensively employed to assess the quality of muscle foods. For instance, Fourier-transform infrared spectroscopy (FTIR) has been successfully employed for meat quality and fraud detection [8,9,10]. Similarly, Raman spectroscopy has also shown its potential to provide structural information about muscle proteins [11,12]. Likewise, visible spectroscopy can be used as a non-invasive method for tissue characterization [13]. Furthermore, fluorescence spectroscopy can be employed for quality evaluation of meat and meat-based products [7,14,15,16,17]. Recently, near-infrared spectroscopy and hyperspectral imaging have also been investigated for non-destructive prediction of quality and compositional analysis of meat [18,19,20,21,22] and for the determination of adulterants in meat [23].

The application of visible–near-infrared (Vis–NIR) spectroscopy directly on the meat carcass is advantageous because it does not require the preparation of the sample before analysis and it is applicable to the prediction of quality online using a fibre-optic probe. Vis–NIR spectra would allow a timely prediction of meat quality traits which could potentially assist the processors to sort out and classify carcasses accordingly. Consequently, the objective of this study was to model the physico-chemical parameters of beef quality including ultimate pH, colour, cook loss and drip loss, using visible–near infrared spectroscopic profiles collected at various time points post-mortem. Spectra were recorded by directly applying a fibre-optic probe on the exposed surface of the carcass (neck and rump) in the abattoir (1 h and 2 h post-mortem), on the cut surface of carcass in the abattoir immediately and one hour after quartering (24 h and 25 h post-mortem), on the cut surface of musculus longissimus thoracis et lumborum (LTL) in the laboratory (48 h and 49 h post-mortem).

## 2. Materials and Methods

### 2.1. Animals and Meat Samples Preparation

A total of three 368 cross-bred beef animals, reared under the same environmental and feeding conditions in the Irish Cattle Breeders Federation Tully Progeny Test Centre were used for the study over a time span of 18 months. The animals were slaughtered in 10 batches from February 2014 until September 2015 in a commercial EU-licensed abattoir in Ireland. All carcasses were quartered at the 8th rib on pistol hind 24 h post-mortem, and the loins muscles were deboned 48 h post-mortem. A total of 12 steaks (2.54 cm thickness) were sliced from the right side of the LTL muscle and vacuum-packed at 4 °C for further analysis. Then, 48 h post-mortem, the loins were transported from the factory to the Teagasc Food Research Centre, Ashtown for further analysis. 

### 2.2. Spectra Collection

Vis–NIR spectra were collected using a portable Vis–NIR spectrophotometer (ASD Inc., Boulder Colorado, CO, USA) with detection waveband range from 350 to 2500 nm, using the Indico Pro program. A high-intensity contact probe was used to transmit the light reflected from the surface of the carcass to the internal detector. Prior to spectral acquisition, the instrument was calibrated using a Spectralon tile as the white reference. Spectra were collected on the day of slaughtering from neck and rump, specifically, one hour after slaughtering (1 h post-mortem) and two hours after slaughtering (2 h post-mortem). Spectra were also collected from the quartered surface of the carcass (5th rib) at the time of quartering (24 h and 25 h post-mortem, after 1 h blooming in the chill room). Spectra were also collected from the LTL muscle in the laboratory (48 h and 49 h post-mortem, after 1 h blooming in the chill room). The spectra were collected in triplicate from three representative sites of the transverse surface of the LTL muscle (the method was described in detail [24]). For each of these three scans, 20 spectra were automatically collected by the instrument consecutively and averaged to reduce noise. Spectral data were exported as a JCAMP file to The Unscrambler X version 10.3 (CAMO ASA, Oslo, Norway) for further chemometric analysis.

### 2.3. Chemical and Physical Analyses

#### 2.3.1. Ultimate pH (pHu)

The ultimate pH was determined in 366 beef carcasses between the 12th and the 13th rib (48 h post-mortem). A portable pH meter was employed (Hanna Instrument HI 9126, Woonsocket, RI, USA) to record both the pH and the temperature. Each sampling day, the pH meter was calibrated with standardized buffers at pH 7.0 and pH 4.0. 

#### 2.3.2. Colour

The equipment employed was the UltraScan^®^ PRO with a dual-beam xenon flash spectrophotometer (λ: 350–1050 nm, Δλ: 5 nm, D65, 8°). The steaks (48 h post-mortem) obtained from LTL muscles were wrapped in oxygen-permeable transparent film and keptfor 1 h of blooming before being measured with the uppermost side placed to the light. The spectrophotometer was calibrated with a black and white baseline. L* (brightness) (varies from 100 for perfect white to 0 for black), a* (redness) (a negative value indicates green, while a positive value indicates red) and b* (yellowness) (a negative value indicates blue, and a positive value indicates yellow) are the coordinates which describe the colour of meat. EasyMach QC software (Hunter Associates Laboratory, Inc., Reston, VA, USA) was used, and the colour coordinates values were obtained as the average of three measurements performed on different locations of each LTL muscle slice.

#### 2.3.3. Cooking Loss Percentage

The samples (14-day-aged steaks) were cooked in a circulating water bath to an internal temperature of 70 °C. The temperature was monitored continuously during the cooking process until a plateau was achieved at 70 °C, using a temperature probe (Eirelec Ltd, Dublin, Ireland) inserted into the geometric centre of the steak. Samples were weighted before and after cooking in order to determine the cooking loss percentage.

Cook-loss (%) = (raw weight − cooked weight)/raw weight × 100(1)

#### 2.3.4. Drip Loss

Drip loss was determined on LTL muscles, 48 h post-mortem. A meat slice of approximately 100 g weight (2.5 cm thickness, 7.5 cm length, 5.0 cm width) was cut from the LTL muscle and hung in the chill room at 4 °C. The samples were weighed after 96 hours, and the drip loss percentage was calculated [25].

Drip-loss (%) = (Initial weight − Final weight)/initial weight × 100(2)

### 2.4. Data Analysis

Partial least-squares regression was performed using The UNSCRAMBLER program (version 8.5.0, Camo, Trondheim, Norway). After visual inspection, the detection of anomalous spectra was accomplished using the H-statistic, which indicates how different a sample spectrum is from the average spectrum of the set [26]. A sample with an H statistic of standardized units from the mean spectrum was defined as a global H outlier and was eliminated from the population. Baseline correction was applied to the spectra because the NIR spectra are affected by light scatter and path-length variation, and pre-treatments of the spectral data improve the accuracy of calibration. In these cases, spectral data pre-treatments such as standard normal variate (SNV) were applied to the spectra to reduce the noise and light scattering effects. Partial least-squares (PLS) regression was used for predicting the chemico-physical properties using Vis–NIR spectra as independent variables. Internal full cross-validation was performed to avoid overfitting the PLS equations; thus, the optimal number of factors in each equation was determined as the number of factors after which the standard error of cross-validation no longer decreased substantially. The accuracy of prediction was evaluated in terms of coefficient of determination (R^2^C and R^2^CV) and root-mean-square error of calibration (RMSEC) and cross-validation (RMSECV).

## 3. Results and Discussion

### 3.1. Spectral Profiles

**Figure 1:**
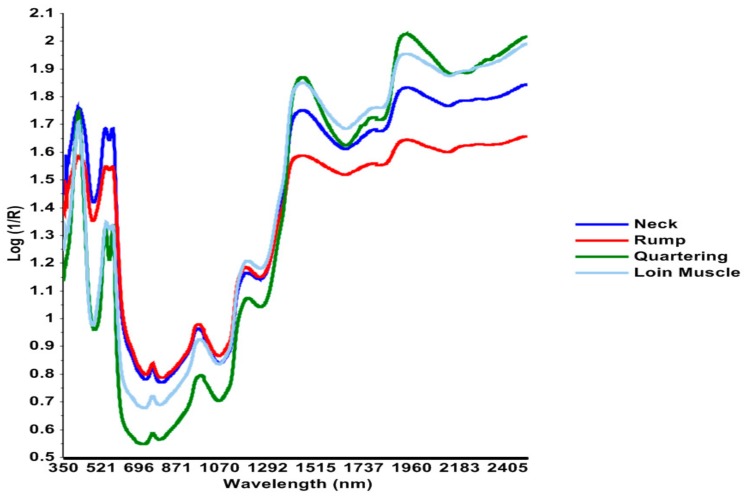
Average visible–near-infrared (Vis–NIR) spectra measured from neck and rump (1 h post-mortem), on quartering from the musculus longissimus thoracis et lumborum (LTL) muscle (24 h post-mortem) and from the loin muscle (48 h post-mortem).

Figure 1 illustrates the average Vis–NIR spectra measured for neck and rump (1 h post-mortem), quartered LTL muscle (24 h post-mortem) and loin muscle (48 h post-mortem). A remarkable difference was seen in the absorbance between the mean spectra of the sites. The variation in the spectra was potentially due to differences in the time of spectral collection (1 h to 48 h post-mortem) and type of muscle. Neck and rump showed lower absorbance as compared to the quartered surface of the LTL muscle and loin muscle in the 1300–2500 nm range. The spectra that were collected during quartering (24 h post-mortem) showed resemblance with the spectra that were collected from the LTL muscle (48 h post-mortem). This is due the fact that, although the spectra were collected in 24 h intervals, they were from the same muscle. Various spectral absorbance bands were identified from the average Vis–NIR range of the analysed samples. These bands could be easily identified by visual inspection of the wavebands at which the highest absorbance values were found. Spectral collection from the neck and quartered surface showed intense peaks at 415 nm and in the 540–580 nm range. Less intense peaks at 1445 nm and 1940 nm were observed for the neck and rump. However, spectral data collected at the time of quartering and in loin muscles showed an inverse pattern of peaks in this region. These wavelengths correspond to different specific functional bonds. Prominent peaks could be seen at 415 nm, 540–580 nm, 1449 nm and 1933 nm [27]. The visual spectral features were analogous to the findings of Andrés et al. [28].

**Figure 2:**
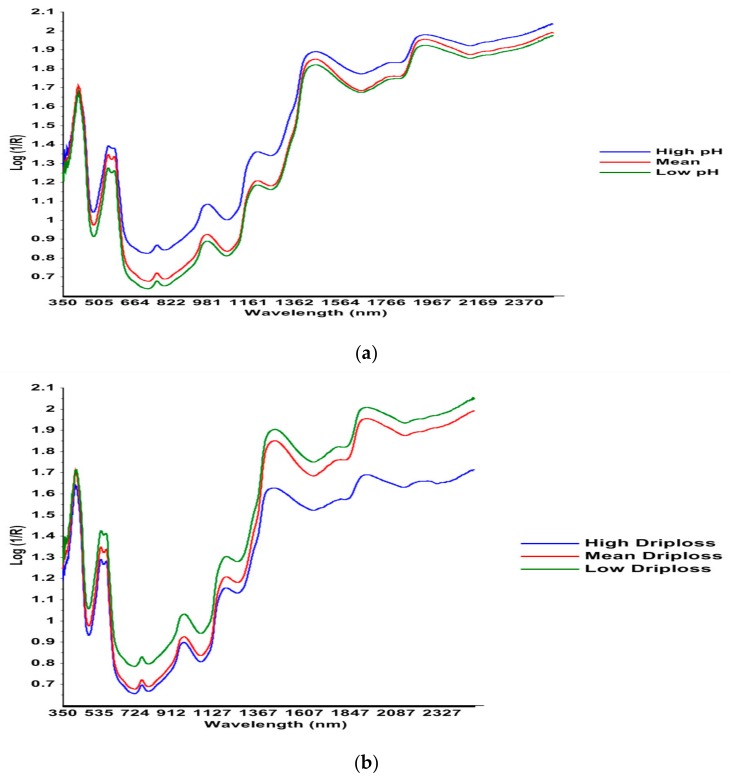
Average Vis–NIR spectra recorded from the LTL muscle (48 post-mortem). (**a**), Spectra of 10 high-ultimate-pH and 10 low-ultimate-pH carcass samples, (**b**) spectra of 10 high-drip-loss and 10 low-drip-loss carcass samples compared to the average spectrum of all samples.

The average spectrum of 10 samples with high ultimate pH values and that of 10 spectrum with low ultimate pH values, compared to the average spectrum of all samples, determined using Vis–NIR spectroscopy, are shown in Figure 2a. The graph depicts higher absorbance value for the samples having low ultimate pH in comparison with those having high ultimate pH. Low-pH-sample spectra showed peak intensity closer to that of the samples with average values of pH, in agreement with the fact that most of the samples in our study had relatively lower ultimate pH and samples with high ultimate pH were more unusual. The absorbance bands at 415 nm and 546 nm were quite prominent and correspond to myoglobin [29]. These results show the efficiency of Vis–NIR spectroscopy in analysing the variation of the ultimate pH of beef.

Figure 2b shows the spectral results concerning the percentage of drip loss for 10 high-drip-loss and 10 low-drip-loss samples. Differences in the absorbance values of the mean samples can be seen. High-drip-loss samples showed less abundant peaks in the spectral range of 1200–2400 nm that corresponds to moisture content [18]. Low-drip-loss samples showed peak intensities closer to those of samples with average drip loss values, especially in the 1200–2400 nm range, showing that most of the samples in our study had relatively low drip loss.

These figures suggest that the absorbance value may be closely proportional to the drip loss, supporting the premise that Vis–NIR spectroscopy has relevance for online prediction of drip loss percentage in post-mortem beef samples

### 3.2. Descriptive Statistics of Beef Samples

Ranges, means, standard deviations and coefficient of variances of physico-chemical traits of all beef samples are given in Table 1. The results depicted a range of 5.16–6.91 for the ultimate pH of the beef samples stored for 48 h. An ultimate pH falling between 5.4–5.8 would be considered normal for beef, with higher values potentially corresponding to a dark, firm and dry phenotype, and lower values potentially presenting issues about their water-holding capacity [30]. Overall, however, the ultimate pH data showed a relatively low coefficient of variance (CV) of 3.23%. A relatively low variation in ultimate pH has been reported in several studies in the past by scientists who investigated the use of near-infrared spectroscopy in beef [28,31,32], chicken [33] and pork meat [34]. Here, it is possible that some outliers could contribute to the range measured, and these outliers were identified during data processing and removed from the data set. The ranges for L*, a* and b* colours were from 35.17 to 50.91, from 8.20 to 19.80 and from 6.34 to16.94, respectively. The descriptive statistical analyses of drip loss (%) and cooking loss (%) are also summarized in Table 1. The average values of drip loss and cook loss were 2.94% and 31.22%, respectively. Drip loss showed considerably higher variability (CV = 51.02%) compared to cook loss (CV = 9.55%). Data obtained from traditional laboratory methods showed that the dataset exhibited relatively low variability in certain parameters and higher variability in others. Overall, the values of the studied parameters were in line with previous findings [30].

### 3.3. Prediction of the pH Ultimate Values of Beef from Vis–NIR Spectra

Prediction models were developed to predict the ultimate pH (48 h post-mortem) of LTL muscles from the spectra collected from neck and rump on the day of slaughtering (1 h and 2 h post-mortem), at quartering time (24 h and 25 h post-mortem) and from LTL muscles (48 h and 49 h post-mortem). The results of prediction of the ultimate pH of beef samples, including the coefficient of determination of calibration (R^2^C), the root-mean-square error of calibration (RMSEC), the coefficient of determination of cross-validation (R^2^CV) and the root-mean-square error of cross validation (RMSECV), are presented in Table 2. Poor predictions were observed from the spectra collected from beef neck (1 h post-mortem: R^2^CV = 0.22 and RMSECV = 0.18; 2 h post-mortem: R^2^CV = 0.16 and RMSECV = 0.21) and rump (1 h post-mortem: R^2^CV = 0.04 and RMSECV = 0.17; 2 h post-mortem: R^2^CV = 0.23 and RMSECV = 0.20), but good predictions were recorded from the spectra collected at the time of quartering (24 h post-mortem: R^2^CV = 0.66 and RMSECV = 0.15). It is interesting to note that when the spectral data collected at the time of quartering (24 h post-mortem) were subjected to baseline correction and SNV correction, the prediction results improved significantly (R^2^CV = 0.91 and RMSECV = 0.17). However, these spectral corrections did not bring any improvement when applied to the spectral data collected from neck and rump (1 h and 2 h post-mortem). Vis–NIR spectra collected from LTL muscles showed good prediction of the ultimate pH (48 h post-mortem: R^2^CV = 0.67 and RMSECV = 0.11; 49 h post-mortem: R^2^CV = 0.73 and RMSECV = 0.11). When baseline and SNV corrections were applied to the spectral data collected from LTL muscle, they improved the PLS model (R^2^CV = 0.96 and RMSECV = 0.25). A previous study also showed that the prediction of pH is possible using Vis–NIR spectroscopy, despite the narrow ultimate pH range of the sample. The findings of the current investigation are in line with the work of De Marchi [27], who found similar results while developing prediction models of beef quality traits using Vis–NIR spectroscopy. These results demonstrate that the prediction of the ultimate pH is possible in the meat industry immediately after quartering or for LTL muscles using Vis–NIR spectra.

### 3.4. Prediction of Drip Loss

Water represents about 75% of the total fresh weight of meat. Vis–NIR spectral data showed the absorbance of O–H bonds at 1450 and 1940 nm. Drip loss percentage was measured for LTL muscles 48 h post-mortem. The results of the PLS model of drip loss from the spectral data collected from various regions (neck, rump, quartered surface and LTL muscle) of beef carcass during post-slaughtering storage are plotted in Table 3. The results revealed lower coefficients of determination of cross-validation for neck (1 h post-mortem: R^2^CV = 0.20; 2 h post-mortem: R^2^CV = 0.10) and rump (1 h post-mortem: R^2^CV = 0.17; 2 h post-mortem: R^2^CV = 0.09), whilst high coefficients of determination of cross-validation were recorded for quartered surface (24 h post-mortem: R^2^CV = 0.51) and LTL muscle (48 h post-mortem: R^2^CV = 0.99). After applying baseline corrections and SNV, the PLS model results from the spectral data collected during quartering were improved (24 h post-mortem: R^2^CV = 0.82). The values of RMSECV in all PLS models were between 1.12 and 1.43, which shows that the model is relatively good for the prediction of drip loss from spectral datasets. These findings have confirmed the potential of Vis–NIR spectroscopy to predict the drip loss percentage of beef during pre-rigor storage. The corollaries of the present study are in accordance with the findings of Prieto et al. [35], who investigated the capability of Vis–NIR reflectance spectroscopy for the prediction of the physical, chemical and sensory quality of beef. It was found that the best prediction model was achieved when spectral data were collected 24 h post-mortem at the quartering stage and 48 h post-mortem from the LTL muscle. This might be due to the fact that the actual drip loss of the samples was also analysed on LTL muscles. Therefore, our results indicate that the actual drip loss of LTL muscles can be predicted from the spectra obtained during quartering or from the spectra collected on the surface of LTL muscles.

### 3.5. Cooking Loss Measurement 

The results presented in Table 4 show that the Vis–NIR spectral data provided moderate prediction accuracy for the determination of cooking loss from the PLS model. Poor coefficients of determination of cross-validation were obtained for neck (1 h post-mortem: R^2^CV = 0.19, RMSECV = 2.70; 2 h post-mortem: R^2^CV = 0.09, RMSECV = 3.41), rump (1 h post-mortem: R^2^CV = 0.14, RMSECV = 2.79; 2 h post-mortem: R^2^CV = 0.28, RMSECV = 2.99), quartered surface (24 h post-mortem: R^2^CV = 0.25, RMSECV = 2.59; 25 h post-mortem: R^2^CV = 0.22, RMSECV = 2.64) and LTL muscles (48 h post-mortem: R^2^CV = 0.43, RMSECV = 2.27; 49 h post-mortem: R^2^CV = 0.45, RMSECV = 2.23). However, it is interesting to note that a lower value of RMSECV was obtained for the PLS models, showing the potential of spectral data to predict cooking loss. Models were also built with baseline and SNV corrections, but the results did not improve significantly in terms of R^2^CV. These spectral corrections had little or no effect on the regression results. According to the literature available, the prediction of cooking loss using Vis–NIR spectroscopy has not been very successful. Our findings are in accord with those of Prieto et al. [35], but nevertheless show greater accuracy than the results presented by De Marchi et al. [36].

### 3.6. Prediction of Colour Parameters

No significant variations were found in the colour of different analysed samples [37]. The results of the PLS models for spectral data collected from neck, rump, quartered surface and LTL muscles of beef using Vis–NIR spectroscopy and colour parameters are shown in Table 5. Higher RMSECV values for L* colour were obtained for the neck (2.20–2.30) and rump (2.18–2.26) as compared to quartered surface (1.95–1.99) and LTL muscle sections (1.76–1.80). A relatively good prediction was recorded for a* and b* colours, although with lower coefficients of determination of calibration (R^2^C = 0.02–0.41 for a*; R^2^C = 0.00–0.46 for b*), but good values of RMSECV (0.75–1.82 for a*; 1.38–1.83 for b*). The NIR spectra correspond to the overtones and combinations of fundamental vibrations of C–H, N–H, O–H and S–H functional groups and provide information about the chemical composition of a sample. These findings are in harmony with the work done by Williams and Norris [26], who suggested that spectral measurements along with chemometric models can be used for the prediction of colour in meat. In our research, we were able to find good prediction models based on lower RMSEC and RMSEV for colour measurement from Vis–NIR data. However, lower values for R^2^C and R^2^CV were obtained, which might be due to colour variation across different areas of the same slice of meat. Ideally, the sample point location in the muscle that is used for colour measurement through traditional methods would be the same as the sample point location that is used for spectral collections; this could be important in developing a regression model. However, our data were averages of three spectra and three colour measures, hence, they should be reflective of the average colour and average spectra of the piece.

## 4. Conclusions

This research work demonstrates the potential of Vis–NIR infrared spectroscopy to predict certain quality parameters of beef. The results are quite promising, showing the potential of this method for online prediction of quality traits in the meat industry. In the case of spectral pre-treatments, baseline correction (BS), SNV and their combinations served as the best pre-treatments, enhancing model accuracy in many cases. Reliable and accurate PLS models were obtained for the ultimate pH for spectra recorded immediately after slaughtering, at quartering time (24 h post-mortem) and from the LTL muscle (48 h post-mortem), whereas for drip loss, the only reliable model was achieved at quartering time (24 h post-mortem). On the other hand, the PLS models for cook loss gave moderate results for the spectra collected on the cut face of the LTL muscle 49 h post-mortem. Similarly, the cut face of the LTL muscle was also observed to be the best regions for colour measurements (48 h and 49 h post-mortem), since the accuracy of the PLS models in this muscle was significantly higher as compared to that for other regions of the carcass and time points. Taken together with findings from our earlier work [24], it can be deduced from the study that Vis–NIR spectroscopy can be a useful tool for the on-site prediction of certain meat quality parameters using different regions of the carcass and different time points, and therefore, this technology has some potential for the beef sector, both for meat management systems of processors and in livestock breeding programs.

## Figures and Tables

**Table 1:** Ranges, mean, standard deviations and coefficient of variance of physico-chemical traits of all beef samples.

Parameter	*n*	Range	Mean	SD	CV (%)
Ultimate pH	366	5.16–6.91	5.58	0.18	3.23
Drip Loss (%)	224	0.29–9.04	2.94	1.50	51.02
Cook Loss (%)	293	16.81–38.02	31.22	2.98	9.55
Colour L*	368	35.17–50.91	42.99	2.44	5.68
Colour a*	368	8.20–19.80	14.30	1.82	12.73
Colour b*	368	6.34–16.94	11.40	1.76	15.44

*n*: number of samples; SD: standard deviation; CV: Coefficient of variance; L*: brightness; a*: redness; b*: yellowness.

**Table 2:** Prediction of pH ultimate of beef samples using Vis–NIR spectra.

pH Ultimate	Math Treatment	*n*	F	R^2^C	RMSEC	R^2^CV	RMSECV
Neck 1 h-PM	Log (1/R)	357	1	0.13	0.18	0.22	0.18
Neck2 h-PM	Log (1/R)	153	4	0.22	0.20	0.16	0.21
Rump 1 h-PM	Log (1/R)	358	6	0.11	0.16	0.04	0.17
Rump 2 h-PM	Log (1/R)	153	4	0.30	0.19	0.23	0.20
Quartering 24 h-PM	Log (1/R)	361	4	0.74	0.13	0.66	0.15
Quartering 24 h-PM	BS + SNV	361	6	0.92	0.34	0.91	0.17
Quartering 25 h-PM	Log (1/R)	366	9	0.36	0.14	0.22	0.16
LTL muscle 48 h-PM	Log (1/R)	223	6	0.71	0.10	0.67	0.11
LTL muscle 48 h-PM	SNV	223	5	0.96	0.33	0.96	0.25
LTL muscle 49 h-PM	Log (1/R)	191	8	0.80	0.09	0.73	0.11

PM: Post-mortem; *n*: number of total samples; F: number of partial least-squares (PLS) latent variables; R^2^C: coefficient of determination of calibration; RMSEC: root-mean-square error of calibration; R^2^CV: coefficient of determination of cross-validation; RMSECV, root-mean-square error of cross validation; Log (1/R): raw absorbance data; BS: baseline correction; SNV: standard normal variate.

**Table 3:** Prediction of drip loss (%) of beef samples using Vis–NIR spectra.

Drip Loss (%)	Math Treatment	*n*	F	R^2^C	RMSEC	R^2^CV	RMSECV
Neck 1 h-PM	Log (1/R)	213	4	0.24	1.32	0.20	1.36
Neck 2 h-PM	Log (1/R)	153	4	0.17	1.10	0.10	1.15
Rump 1 h-PM	Log (1/R)	212	3	0.18	1.37	0.17	1.38
Rump 2 h-PM	Log (1/R)	153	4	0.17	1.10	0.09	1.16
Quartering 24 h-PM	Log (1/R)	214	4	0.54	1.22	0.51	1.34
Quartering 24 h-PM	BS + SNV	214	3	0.82	1.44	0.82	1.43
Quartering 25 h-PM	Log (1/R)	219	4	0.22	1.32	0.17	1.37
LTL muscle 48 h-PM	Log (1/R)	224	2	0.99	0.11	0.99	1.12
LTL muscle 49 h-PM	Log (1/R)	192	7	0.43	1.12	0.32	1.24

*n*: number of total samples; F: number of PLS latent variables; R^2^C: coefficient of determination of calibration; RMSEC: root mean square error of calibration; R^2^CV: coefficient of determination of cross-validation; RMSECV, root mean square error of cross validation; Log (1/R): raw absorbance data; BS: baseline correction; SNV: standard normal variate.

**Table 4:** Prediction of cook loss (%) of beef samples using Vis–NIR spectra.

Cook Loss (%)	Math	*n*	F	R^2^C	RMSEC	R^2^CV	RMSECV
Neck 1 h-PM	Log (1/R)	286	8	0.34	2.44	0.19	2.70
Neck 2 h-PM	Log (1/R)	81	2	0.18	3.20	0.09	3.41
Rump 1 h-PM	Log (1/R)	285	8	0.26	2.50	0.14	2.79
Rump 2 h-PM	Log (1/R)	81	8	0.58	2.28	0.28	2.99
Quartering 24 h-PM	Log (1/R)	293	10	0.43	2.25	0.25	2.59
Quartering 25 h-PM	Log (1/R)	293	10	0.41	2.28	0.22	2.64
LTL muscle 48 h-PM	Log (1/R)	151	6	0.51	2.00	0.43	2.27
LTL muscle 49 h-PM	Log (1/R)	150	6	0.53	2.06	0.45	2.23

*n*: number of total samples; F: number of PLS latent variables; R^2^C: coefficient of determination of calibration; RMSEC: root mean square error of calibration; R^2^CV: coefficient of determination of cross-validation; RMSECV, root mean square error of cross validation; Log (1/R): raw absorbance data.

**Table 5:** Prediction of colour parameters of beef samples using Vis–NIR spectra.

Colour L*	Math	*n*	F	R^2^C	RMSEC	R^2^CV	RMSECV
Neck 1 h-PM	Log (1/R)	361	6	0.24	2.11	0.18	2.20
Neck 2 h-PM	Log (1/R)	155	1	0.03	2.30	0.01	2.30
Rump 1 h-PM	Log (1/R)	360	7	0.29	2.05	0.20	2.18
Rump 2 h-PM	Log (1/R)	155	9	0.37	1.88	0.11	2.26
Quartering 24 h-PM	Log (1/R)	368	9	0.42	1.85	0.33	1.99
Quartering 2 5 h-PM	Log (1/R)	368	8	0.42	1.84	0.36	1.95
LTL muscle 48 h-PM	Log (1/R)	224	8	0.60	1.55	0.49	1.76
LTL muscle 49h-PM	Log (1/R)	191	7	0.53	1.65	0.44	1.80
Colour a*							
Neck 1h-PM	Log (1/R)	361	8	0.21	1.62	0.08	1.75
Neck 2 h-PM	Log (1/R)	155	5	0.18	1.67	0.11	1.75
Rump 1 h-PM	Log (1/R)	360	1	0.02	1.81	0.09	1.82
Rump 2 h-PM	Log (1/R)	155	1	0.06	1.78	0.06	1.81
Quartering 24 h-PM	Log (1/R)	368	7	0.23	1.58	0.15	1.67
Quartering 25 h-PM	Log (1/R)	368	3	0.09	1.72	0.07	0.75
LTL muscle 48 h-PM	Log (1/R)	224	6	0.41	1.35	0.32	1.46
LTL muscle 49 h-PM	Log (1/R)	191	4	0.31	1.37	0.28	1.41
Colour b*							
Neck 1 h-PM	Log (1/R)	361	1	0.00	1.76	NA	1.79
Neck 2 h-PM	Log (1/R)	155	1	0.01	1.80	NA	1.83
Rump 1 h-PM	Log (1/R)	360	1	0.00	1.76	NA	1.79
Rump 2 h-PM	Log (1/R)	155	1	0.01	1.80	NA	1.82
Quartering 24 h-PM	Log (1/R)	368	8	0.30	1.47	0.19	1.57
Quartering 25 h-PM	Log (1/R)	368	9	0.34	1.43	0.20	1.57
LTL muscle 48 h-PM	Log (1/R)	224	6	0.46	1.30	0.39	1.39
LTL muscle 49 h-PM	Log (1/R)	191	5	0.40	1.33	0.32	1.41

*n*: number of total samples; F: number of PLS latent variables; R^2^C: coefficient of determination of calibration; RMSEC: root mean square error of calibration; R^2^CV: coefficient of determination of cross-validation; RMSECV, root mean square error of cross validation; Log (1/R): raw absorbance data.
